# A Chemical Template for Synthesis of Molecular Sheets of Calcium Carbonate

**DOI:** 10.1038/srep25393

**Published:** 2016-05-05

**Authors:** Ina Rianasari, Farah Benyettou, Sudhir Kumar Sharma, Thomas Blanton, Serdal Kirmizialtin, Ramesh Jagannathan

**Affiliations:** 1 Engineering Division, New York University, Abu Dhabi, UAE; 2 Chemistry Program, New York University Abu Dhabi, Abu Dhabi, UAE; 3 International Centre for Diffraction Data, Newtown Square, PA, 19023, USA

## Abstract

Inspired by the discovery of graphene and its unique properties, we focused our research to develop a scheme to create nacre like lamellar structures of molecular sheets of CaCO_3_ interleaved with an organic material, namely carbon. We developed a facile, chemical template technique, using a formulation of poly(acrylic) acid (PAA) and calcium acetate to create lamellar stacks of single crystal sheets of CaCO_3_, with a nominal thickness of 17 Å, the same as a unit-cell dimension for calcite (c–axis = 17.062 Å), interleaved with amorphous carbon with a nominal thickness of 8 Å. The strong binding affinity between carboxylate anions and calcium cations in the formulation was used as a molecular template to guide CaCO_3_ crystallization. Computational modeling of the FTIR spectra showed good agreement with experimental data and confirmed that calcium ions are bridged between polymer chains, resulting in a net-like polymer structure. The process readily lends itself to explore the feasibility of creating molecular sheets of other important inorganic materials and potentially find applications in many fields such as super capacitors and “low k di-electric” systems.

In nature, it is well known that interactions of organic matrices and inorganic compounds of biological origin result in self-organized, hierarchical structures^1^,^2^,^3^. In order to achieve such directed self-assembly nanostructures, a number of possible mechanisms have been suggested in literature^4^,^5^,^6^,^7^,^8^ but synthetic and bio-inspired methods have been found to be more suitable for the fabrication of biomimetic structures. For example, self-assembled monolayers of functionalized alkanethiols on gold and silver substrates have been used for selectively directing the orientation of CaCO_3_ crystal growth in 2-dimensions^9^,^10^. Organic matrices composed of macromolecules such as proteins and synthetic polymers are well known to influence morphologies and polymorphism over a 3-dimensional scale^11^,^12^,^13^,^14^,^15^,^16^. Among the various bio-inspired materials, nacre, which is essentially a lamellar stack of CaCO_3_ nano-sheets, interleaved with organic material, has long intrigued researchers because of its special properties resulting in designing synthetic routes for nacre like structures^17^. Inspired by the discovery of graphene and its unique properties, in our research, we have attempted to extend the focus to create lamellar structures of molecular sheets of CaCO_3_ interleaved with organic material. While we have used CaCO_3_ as a model compound, our aim is to develop a generic synthetic procedure that could be extended as a potential platform for the development of a broader class of materials.

Using CaCO_3_ as a model compound, and based on a careful review of the literature, we settled on leveraging the strong binding affinity between carboxylate anions and calcium cations using poly(acrylic) acid (PAA) as a molecular template to guide CaCO_3_ crystallization. For our experiments, we chose a lower molecular weight (i.e. M_w_ ~ 8000 Da) PAA for two reasons, namely, higher solubility and more effective tendency to form complexes as compared to the larger molecular weight PAA^18^. We used calcium acetate (Ca-Ac) as a precursor molecule for CaCO_3_ and hypothesized that it would be possible to formulate a mixture of calcium acetate and PAA, in which Ca^2+^ and COO^−^ chelated complexes are primarily attached to each other in a “net-like” structure, while the hydroxyl group connected via hydrogen bonding^19^,^20^,^21^. This would create a pinned, net-like structure of calcium and oxygen atoms extended over a two-dimensional (XY) plane and bridged with PAA backbone along the z-direction (shown in Fig. 1). Solution casting of this formulation on a substrate followed by calcination should result in the crystallization of co-planar, molecular sheets of CaCO_3_ separated by a spacer layer of carbon. The carbon layer should serve as a growth inhibitor for z-directional growth of CaCO_3,_ resulting in the formation of nanostructured molecular sheets. By changing the molar ratio of Ca^2+^/COO^−^, we were able to control the CaCO_3_ growth morphology and consistently produce unit cell dimensional molecular sheets. These molecular sheets were then extensively characterized using both experimental and quantum mechanical methods.

We envision that materials with such a calcite structure would have unique mechanical properties that are significantly superior to the nacre type structures. The frequency of “tablet-sliding” phenomenon that is attributed to the unique mechanical properties of nacre-like materials would be enhanced significantly for these molecular sheets due to reduced mass and the low friction carbon interlayer. We expect the molecular sheets/carbon lamellar structures would be able to release the applied stress at faster rates and therefore be much stronger. We have already reported the evidence of tunable “tablet-sliding” of molecular sheets of Teflon AF-1600 in our earlier publication^22^. Our future expectation is to create formulations that would enable a 3D printing platform for a range of biomaterials^23^,^24^. Moreover, we expect the CaCO_3_ molecular sheets/carbon structures to make significant contributions to the fields of super capacitors and “low k di-electric” systems.

## Results and Discussion

**Figure 2:**
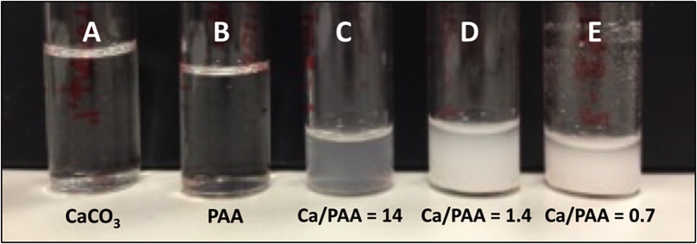
Image of aqueous solutions of (**A**) pristine calcium acetate, (**B**) pristine poly(acrylic) acid, and formulation solutions prepared by mixing calcium acetate (1.25 wt%, 70 mM) and PAA (0.01 wt%) in DI water with three different molar ratios: (**C**) Ca^2+^/COO^−^ = 14, (**D**) Ca^2+^/COO^−^ = 1.4 and (**E**) Ca^2+^/COO^−^ = 0.7. The pristine formulations are transparent without any noticeable cloudiness. Onset of Ca-PAA complex formation is evident by slight turbidity in solution in (**C**). Complex formation is indicated by strong turbidity in solutions (**D**,**E**).

Three different solution formulations along with pristine calcium acetate and poly(acrylic) acid are shown in Fig. 2. It can be seen from Fig. 2A,B that the pristine solutions are transparent without any noticeable cloudiness. PAA is a polycarboxylic acid with pKa of 4.5^25^,^26^,^27^,^28^. Three mixed formulations containing different molar ratios of Ca^2+^/COO^−^ (14, 1.4 and 0.7) are shown in Fig. 2C–E, respectively. The pH of all of these mixed formulations were maintained constant at 7.2 while all of them showed significant solution turbidity, the 0.7 molar ratio formulations were the most turbid. At this pH, the degree of dissociation of the carboxylate group is close to one^25^,^27^. In these formulations the calcium cations (Ca^2+^) of calcium acetate are expected to bind to carboxylate functional groups (COO^−^) of PAA via templated self-assembly process^26^ resulting in Ca-PAA complexes^19^,^27^,^29^. The formation of Ca-PAA complex is indicated by the onset of solution turbidity, observed within few minutes, which is in agreement with previous reports^25^,^30^,^31^,^32^.

### Molecular Sheets of CaCO_3_

**Figure 3:**
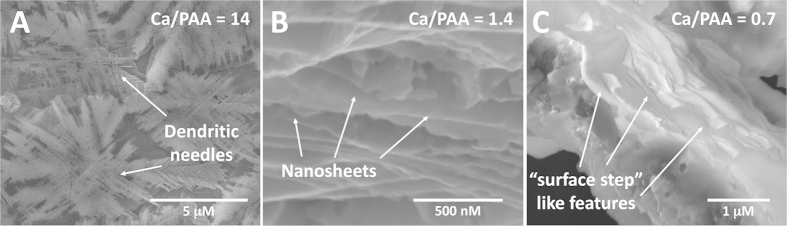
SEM micrographs of complex formed with molar ratio (**A**) Ca^2+^/COO^−^ = 14, showing predominantly “dendritic” like morphology; (**B**) Ca^2+^/COO^−^ = 1.4, showing very thin sheets of CaCO_3_; and (**C**) Ca^2+^/COO^−^ = 0.7, showing thin sheets, resembling surface steps. All solutions were casted on pre-cleaned silicon substrate followed by calcination at 420 °C for about three hours.

**Figure 4:**
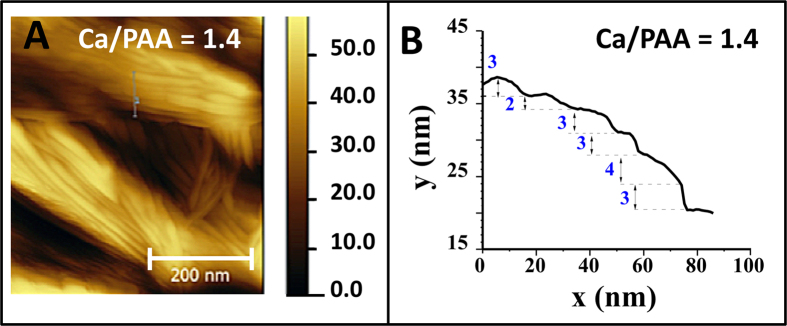
(**A**) Non-contact mode AFM topographic micrograph recorded for CaCO_3_ thin film prepared using molar ratio Ca^2+^/COO^−^ = 1.4. (**B**) Representative height profile of sample Ca^2+^/COO^−^ = 1.4. Confirming the presence of stacked nano-sheets with a height around 2–4 nm indicating one unit cell thick calcite nano-sheets separated by a layer of amorphous carbon.

Solution formulations containing complex precipitates were centrifuged to separate the complex aggregates from the supernatant. Thin films were prepared from solution casting the aggregates on a pre-cleaned silicon substrate and dried in ambient conditions overnight, followed by calcination at 420 °C in air, for about 3 hours. We observed the film formation in Scanning Electron Microscope. For the formulation with the molar ratio of Ca^2+^/COO^−^ = 14, “dendritic” like features were observed (Fig. 3A). For molar ratios of 1.4:1 and 0.7:1, (respectively Fig. 3B,C), lamellar stacks of very thin sheets were observed, consistent with our template hypothesis. Pinned, “net-like” bridges formed in these formulations, when solution casted and calcined resulted in direct crystallization of 2-dimensional CaCO_3_ lamellar sheets^25^. The SEMs micrographs of two control samples, pristine calcium acetate in solution and pristine PAA after calcination at 420 °C are also reported in Fig. S1 (see Supporting Information). The lamellar stacks shown in Fig. 3B were further characterized by AFM and found to contain surface steps with an approximate thickness of 2 to 4 nm (Fig. 4) with a likely implication of unit cell thick calcite sheets separated by a carbon layer.

### Molecular Modeling of PAA/Ca

**Figure 5:**
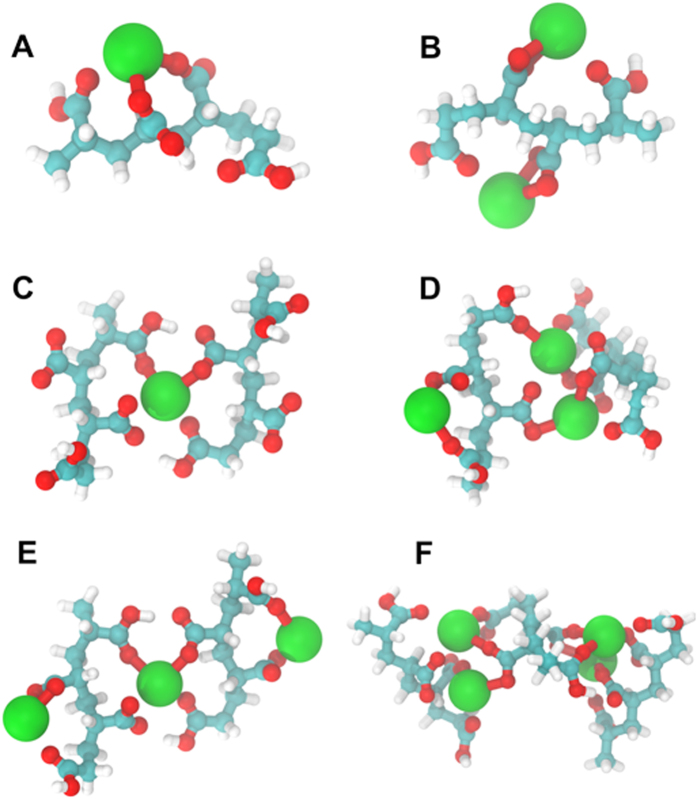
Lowest energy configuration of oligo-acyrylic-acid(s) Complexed with different number of Ca^2+^ calculated by ab initio quantum mechanical approach. (**A**,**B**) are possible configurations of a single chain with one and two calcium ions bonded, suggesting bidendate coordination as the most stable conformation. (**C**) is the lowest energy structure of two chains when there is only one calcium ion (mimicking the low ion condition in experiment); (**D**,**E**) is when the ion is in excess, forming both parallel and rotated chains. (**F**) is the structure of three chains in excess calcium ion. Standard enthalpy of the each structure is tabulated in Table S2 in Supporting Information.

Molecular modeling was carried out to understand the atomic level structure of calcium-induced precipitates. The lowest energy state of oligo-acyrlic acid with one Ca^2+^ ion is shown in Fig. 5A. The mode of binding is bidendate with Ca^2+^ ion bridges two neighboring carboxylate oxygens forming a covalent Ca-O bond. The optimized geometry of (AA_4_)^4−^ with two calcium ions is shown in Fig. 5B. It is to be noted that the mode of coordination could change to another form of bidendate where calcium binding is equally spaced between carbonyl and carboxylate oxygen within a same unit cell.

For deeper insight, we also modeled multi-chain geometries of PAA/Ca. We considered two and three chains and excluded higher orders due to the computational cost. A number of lowest energy states as possible configurations of PAA/Ca is shown in Fig. 5(C–F). In the absence of the metal ion, the main mode of interaction in PAA is hydrogen bonding, between the carboxylate hydrogen and the carbonyl oxygen. Ionization of carboxylate oxygens break the inter-chain hydrogen-bonding network and offer a less stable molecular structure. On the other hand, the structure is stabilized when calcium ions bind to the ionized carboxylates (Table S1).

In contrast to the single chain conformations, our calculations suggest that calcium ions form intra-molecular covalent bonds, resulting in a net-like molecular structure. In low to medium PAA/Ca coordination, the calculated lowest energy state (Fig. 5C) resulted in parallel chains and making the molecular sheet formation viable. In this configuration, the distance between the backbone chains is 8.1 Å; a distance of about half of the periodic spacing observed in our experiments (Fig. 4). The lowest energy state of the two-oligomers with three ions is shown in Fig. 5D,E. The excess calcium ions results in rotating the chains for about 30 degrees and distorts the planarity. The possibility to form a planar structure is still available (Fig. 5D), but is 40.93 kcal/mole higher in energy when compared to the lowest energy configuration (Fig. 5E) suggesting a possible shift of populations from planar to distorted orientations with an increase in calcium ion concentration. Similarly, a distorted structure is obtained for three chains in excess calcium (Fig. 5F). Standard enthalpy of formation of each complex is reported in Table S2. These calculations are in good agreement with our SEM results (shown in Fig. 3), where the molar ratio of Ca^2+^/COO^−^ = 14 shows predominantly needle like morphology, while lower molar ratio of Ca^2+^/COO^−^ = 1.4 and Ca^2+^/COO^−^ = 0.7 (Fig. 3B,C) shows two-dimensional lamellar sheets of CaCO_3_.

### Structural Characterization

**Figure 6:**
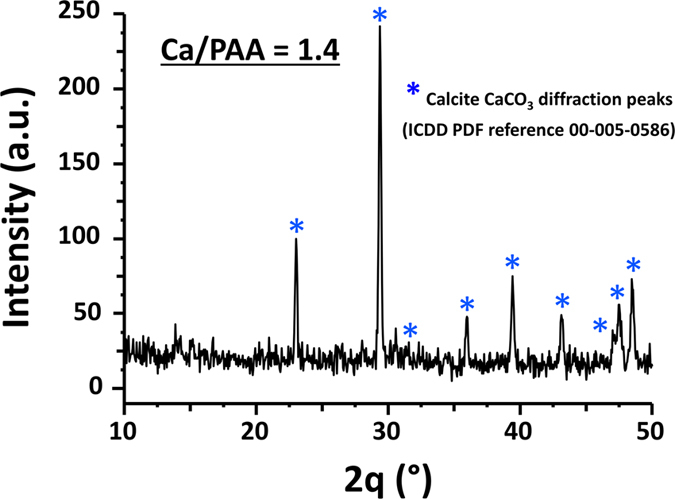
Powder X-ray diffraction pattern collected for a solution casted complex film molar ratio (Ca2+/COO^−^ = 1.4) on silicon after annealing at 420 °C. All the peaks are indexed to the Calcite phase (ICDD PDF reference 00-005-0586) and indicated by a star *.

In Fig. 6 is depicted the XRD pattern for the Ca^2+^/COO^−^ = 1.4 complex film sample after calcination at 420 °C for three hours. The diffraction data confirmed the presence of calcite CaCO_3_ (ICDD PDF reference 00-005-0586). It is important to note that the diffraction peaks are sharp implying that the lamellar stacks are well-aligned. This is consistent with previously reported nanostructured of calcite sheet stacking in nano CaCO_3_ material^32^. The confirmation of nano CaCO_3_ formation with XRD, and lamellar stacks of sheets with an approximate thickness of 2–4 nm from both AFM measurements and SEM images, directly imply that these are molecular sheets of CaCO_3_, consistent with our experimental findings reported in our earlier publication^33^. That is, these are lamellar stacks of well-aligned, two-dimensional, CaCO_3_ single layers with a nominal thickness of 17 Å, the same as a unit-cell dimension for calcite (c–axis = 17.062 Å).

### Spectroscopic Characterizations

**Figure 7:**
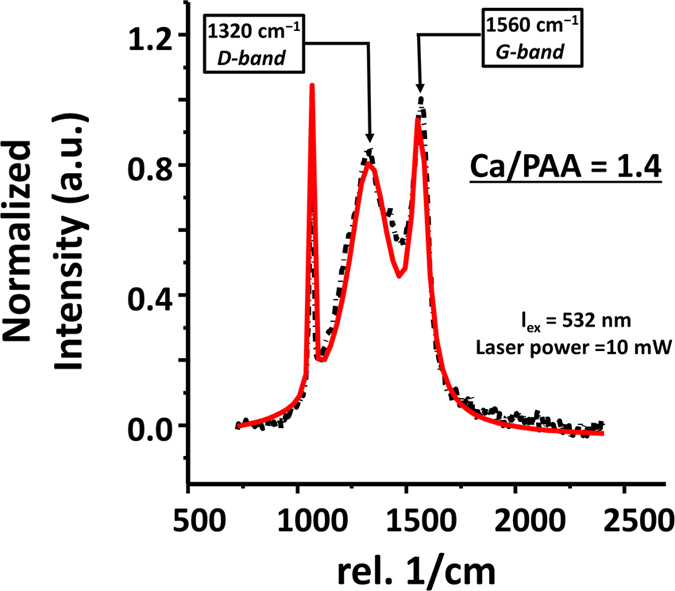
Confocal Raman spectrum for calcinated, complex precipitate sample (Ca^2+^/COO^−^ = 1.4) on silicon, collected using a micro-Raman spectrometer (Witec alpha 300, Germany) at an excitation wavelength of 532 nm. Laser power was kept constant at 10 mW. Typical integration time of 3 s and an average of 20 accumulations were used. The black line indicates the measured Raman spectra and red line shows Lorentzian deconvolution fitted with three peak assignments. The presence of two main peaks located at 1320 cm^−1^ and 1560 cm^−1^ corresponds to D-band and G-bands, respectively, due to amorphous carbon between the thin layers of calcite lamellae.

Confocal Raman recorded for all three different ratios and were found to be identical, we show the Raman spectra of Ca^2+^/COO^−^ = 1.4 as representative of our result (Fig. 7). Raman spectra of the formulations also confirmed the presence of amorphous carbon in the lamellar stacks. Two main peaks at ~1320 cm^−1^ (D-band) and 1560 cm^−1^ (G-band) which are close to the amorphous carbon spectra of 1355 cm^−1^ (D-band) and 1575 cm^−1^ (G-band) were observed^34^,^35^,^36^. The downshift of the G-band to lower frequencies indicate strained carbon-carbon bonds as previously reported in literature^37^,^38^. In addition, all three samples show a broad FWHM of the G-band. Based on the Raman, and AFM data as well as the SEM images, we conclude that the carbon present in this sample is amorphous forming thin layers between calcite lamellae. This nanostructure formation provides an explanation for the absence of carbon by XRD.

**Figure 8:**
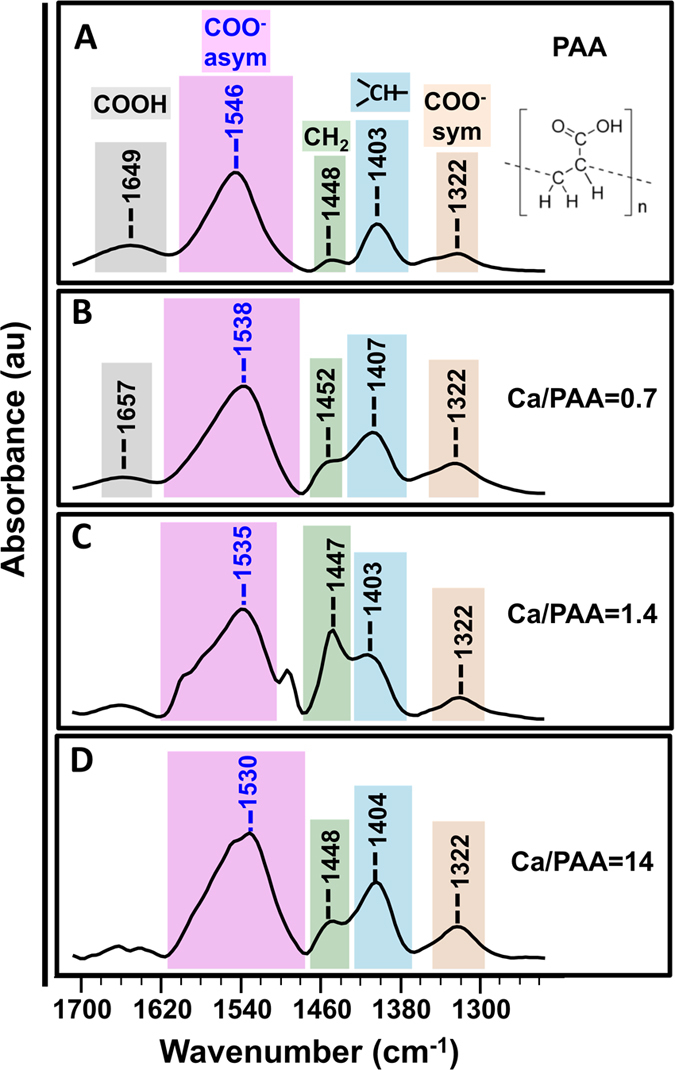
FTIR spectra of dried thin film prepared from the centrifuged and washed complex precipitates on a silicon substrate as a function of the molar ratios of Ca^2+^/PAA. (**A**) PAA in the absence of Ca^2+^ and in the presence of Ca^2+^ in molar ratios of Ca^2+^/PAA (**B**) 0.7, (**C**) 1.4, (**D**) 14. Intensity of the peaks had been normalized using the peak at 1530 cm^−1^ present in all the samples. No. 1035 and 671 cm^−1^ bands were observed in plots (**B**–**D**) indicating the absence of free CaCO_3_ in washed formulations, confirming that the precipitate formation is due to the complexation of the Ca^2+^ and PAA.

**Figure 9:**
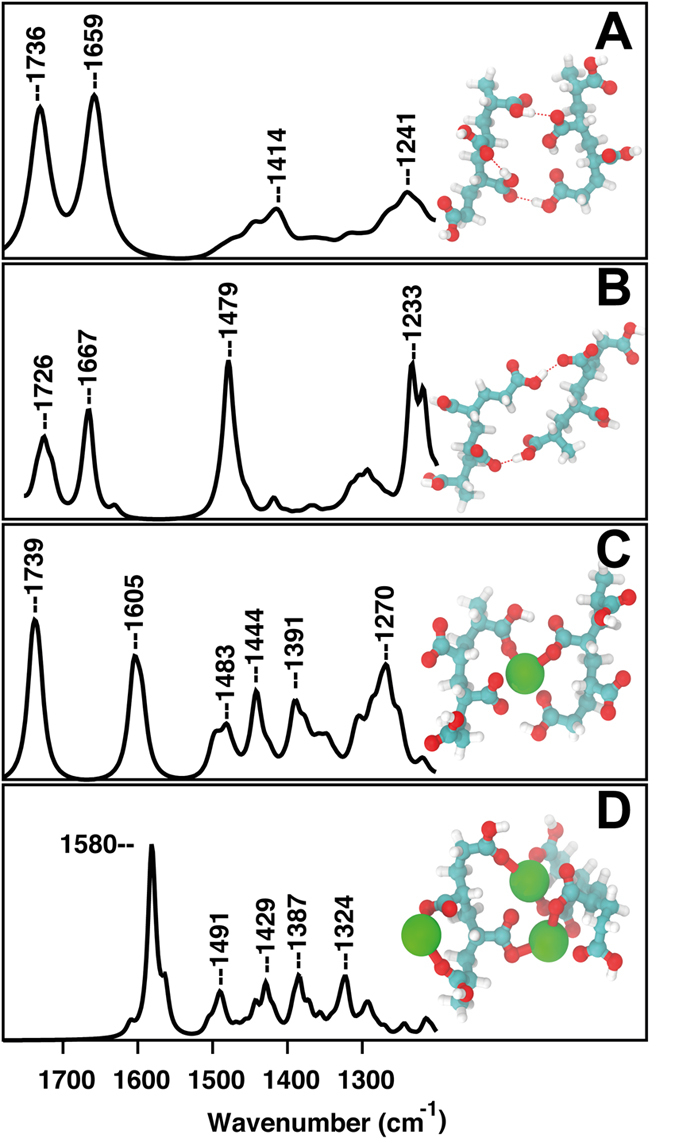
Computed Fourier Transform Infrared Spectra of oligo-acyrlyic chains with different number of Ca^2+^ ions bonded. Shown on the right are model compounds in minimum energy state used for frequency calculations, (**A**) oligomer chains in the absence of calcium ion in comparison to (**B**) ionized chain (**C**) a single calcium ion bound, mimicking the low calcium content and (**D**) three calcium ions bound, mimicking high calcium content. The change in the peak positions upon addition of calcium is in parallel with experimental data suggesting the bidentate coordinated calcium ions bridge between polymer chains.

FTIR spectra for dried PAA (pristine) film and three different washed formulations dried over a silicon substrate are shown Fig. 8. For pristine PAA we used commercial PAA (8000) which is not calcinated. The spectrum of pristine PAA reveals characteristic absorption bands at 1646, 1546 and 1322 cm^−1^ corresponding to ν(COOH), ν(COO^−^ asym), and ν(COO^−^ sym) vibrations, respectively (Fig. 8A). It also consists of two characteristic peaks at 1448 and 1403 cm^−1^ attributed to the ν(CH_2_) and ν (CH) present in the PAA. From the FTIR spectra of the complex formulations, it is clear that these two characteristic PAA absorption bands located at 1448 and 1403 cm^−1^ were unaffected by the addition of CaCO_3_ and are not involved in complexation. Moreover, the characteristic bands of the metal complex at 1035 and 671 cm^−1^ locations are absent from the spectra shown in Fig. 8B–D indicating the absence of free CaCO_3_ in washed formulations^20^,^21^. These observations attest the successful cleaning of the materials and certify that the precipitate formation is due to the complexation of Ca^2+^ and PAA. We observe a slight difference in the intensity of the peaks at 1448 and 1403 cm^−1^ in addition to a new band at about 1490 cm^−1^ during the complexation of PAA with Ca inCa/PAA = 1.4(Fig. 8C). The split in CH peak is also evident in computational IR (Fig. 9), which indicates a split in vibrational modes of CH upon complexation. The split can also be due to the increased thickness of the sample.

It can be seen from Fig. 8B–D that the position of the vibration band of the symmetric carbonyl is independent of the calcium content. While the position of ν(COO^−^ asym) is downshifted with the increase in calcium content. These behaviors are consistent with a bidentate mode of interaction between Ca^2+^ and PAA. Moreover, for all three molar ratios reported here Δν decreases with the increase of calcium content (Table 1). Using the experimental data alone it was not possible to decipher the exact nature of the bidentate binding mode (chelating versus bridging). Quantum mechanical calculations were used to achieve a clearer insight into the mechanism/mode of interaction between the carboxylates with Ca^2+^.

### Computational Modeling of IR Spectra

Similar to experimental FTIR data (shown in Fig. 8), we varied PAA/Ca coordination. The modeled spectra showed a close resemblance with experimental data and a similar change with PAA/Ca coordination. The computational spectra evaluated for neutral two-chain oligo-acrylic acid, ionized chains, one calcium ion bonded (low Ca/PAA ratio), and three calcium ions bonded (high Ca/PAA ratio) (see Fig. 9).

The eigenvectors of the vibrational frequencies were used to assign the molecular modes. The first two peaks correspond to carbon-oxygen vibrational modes in pristine PAA chain (Fig. 9A). The peak centered at 1736 cm^−1^ is due to C=O stretching while the second band is assigned for C-O bonds vibrations. The third peak located at 1414 cm^−1^ corresponds to vibrations of aliphatic chain hydrogens. The last band centered at 1241 cm^−1^ is attributed to -COOH stretch. The peaks that correspond to the vibrational modes of v(COOH) and v(COO- asym) are shifted by %10 from experimental values. The reason for the discrepancy between the experiment and theory is difficult to assess. The empirical scaling of the vibrational frequencies traditionally implemented^39^ provides a global fit to a variety of data sets, so does not guarantee to fit every frequency of PAA successfully. Therefore, in our study we based our conclusions on the modes of vibrations and the relative changes in the peak positions during complex formation.

The modeled spectra for partially ionized polymer are shown in Fig. 9B. Change of –COOH into –COO^−^ resulted in two equal carbon-oxygen band with a force constant value that is between that for C=O and C-O, thus shifted the first peak towards lower wavenumbers by about 10 cm^−1^. The experimental value for the same band is at 1649 cm^−1^ (shown in Fig. 8). In addition, our calculations showed a second peak around 1667 cm^−1^ corresponding to COO^−^ antisymmetric stretch, while the experimental value is around 1544 cm^−1^. The peak at 1479 cm^−1^ is due to aliphatic CH_2_ hydrogens consistent with experimental results with a band at 1448 cm^−1^. The band located at 1233 cm^−1^ attributed to the symmetric COO^−^ stretching with a shift from the experimental value at 1322 cm^−1^. Binding of calcium ions decrease the vibrational frequency of antisymmetric mode values from 1667 → 1605 → 1580 cm^−1^ while the symmetric COO^−^ stretch vibrational frequency values increased from 1233 → 1270 → 1324 cm^−1^. The separation between the vibrational frequency of antisymmetric and symmetric mode becomes smaller (Fig. 9B–D), which is found to be in good agreement with our experimental data shown in Table 1. The close agreement between experimental and simulated FTIR spectra in terms of the peak positions as well as the change in positions as a function of calcium concentration confirmed the bidentate mode of coordination for bridging the carboxylate oxygen with different chains.

## Conclusion

We have used a straightforward, well established chemical template technique and developed a facile formulation process to create lamellar stacks of single crystal sheets of CaCO_3_, with a nominal thickness of 17 Å, the same as a unit-cell dimension for calcite (c–axis = 17.062 Å), interleaved with amorphous carbon. The molecular structure of the complex in different calcium concentrations were investigated by computational modeling of the FTIR spectra and showed a good agreement with experimental data. Our simulations confirmed that the calcium ions bridge between polymer chains, resulting in a net-like polymer structure with periodic spacing of 8.1 Å, which is approximately half of the measured experimental values between the lamellar sheets, suggesting a higher order organization in the experimental results. Further study is underway to understand the intra-molecular organization. We also expect these molecular sheets to have unique mechanical properties and our future experimental plans include studying such properties. We further plan to develop formulations that would enable 3-D printing of these unique materials and thereby creating a viable, bio-materials manufacturing platform. We also believe that the process readily lends itself to explore the feasibility of creating molecular sheets of other important inorganic materials.

## Materials and Method

All chemical reagents are commercially available. Calcium acetate monohydrate (Ca(OAc)_2_H_2_O) and poly(acrylic) acid sodium salt (average MW = 8000 Daltons) were purchased from Sigma Aldrich (USA). The water used in all experiments was prepared in a three-stage Millipore MilliQ plus 185 purification system, and had a resistivity of 18.2 mΩ cm.

### Deposition of CaCO_3_ films

Different precursor solutions were prepared by mixing calcium acetate (1.25 wt%, 70 mM) and PAA (0.01 wt% in DI water) with three different molar ratios of Ca^2+^/COO^−^ (14, 1.4 and 0.7) in aqueous solution. Formulation solutions exhibited turbidity in a matter of a few minutes, due to the formation of complex precipitates, which were centrifuged at 15 K rpm for duration of 2 minute and washed with DI water and separated from the clear supernatant. The pH of the solutions was measured by using a HI 2550 pH meter (Hanna Instrument, USA). Prior to film deposition, Si (100) wafers were diced and cleaned by ultrasonication for 15 min in isopropyl alcohol followed by N_2_ stream drying. An aliquot of 100 μL of complex precipitate was solution-casted on the Si substrates and dried overnight in ambient air. This facilitated the self-assembly process and molecular sheet formation to proceed in an organized manner. Room temperature was chosen for our first series of investigations with an expectation that, if needed, other experimental parametric space (e.g. temperature, molar ratios, molecular weight etc.) would be explored. Subsequently, the precursor solutions of Calcium acetate and PAA were also solution casted onto these substrates and ambient dried for overnight and were followed for calcination at 420 °C for three hours in air. All these solution casted films were dried for overnight in ambient. To decompose Ca(OAc)_2_ to CaCO_3_, these dried solution casted films were calcinated in air at 420 °C for three hours using an oven (Carbolite 1200, UK).

### Characterization

Surface morphology of thin films of CaCO_3_ was characterized by field emission scanning electron microscopy (FE-SEM, FEI, The Netherlands) under high vacuum mode. Film topography was analyzed by atomic force microscopy (AFM, Agilent) using the non-contact mode. Height, phase and amplitude images were acquired simultaneously. Cantilevers (Nanosensors^TM^, Neuchatel, Switzerland) with resonant frequency of 204–497 kHz and force constant of 10–130 Nm^−1^ were used. The set point value was kept at 0.9 V. Gwyddion^TM^ free software (version 2.31) was used for post-processing the acquired topographic scans.

X-ray diffraction measurements were performed with a Panalytical Empyrean system using Cu Kα radiation as the X-ray source. Divergent optics consisted of a parallel mirror with 1/32° divergent slit, 0.04 radian Soller slits and a 10 mm mask. The receiving optics consisted of a 0.27° parallel slit collimator, 0.04 radian Soller slits, and a scintillation detector. Prior to XRD data collection, a precise sample alignment was performed for Z, Omega and Chi viz. Z by blocking half the beam intensity, Omega by low angle reflection and by Chi to maximize intensity, respectively. Two different scan modes such as coupled 2theta-omega scan and 3° grazing angle relative to the sample surface scan were used and XRD pattern collected in grazing angle is reported in this manuscript.

Confocal Raman single spectra for calcinated samples were collected using a micro-Raman spectrometer (Witec alpha 300, Germany) at 532 nm excitation wavelength. Laser power was kept constant at 10 mW. Typical integration time of 3 s and an average of 20 accumulations were used. Individual peak intensities/areas were evaluated by Lorentz peak fitting for three peak locations. The specific nature of binding of Ca^2+^/COO^−^ was further investigated using FTIR measurements. Three molar ratios of calcium acetate and PAA (Ca^2+^/COO^−^ = 0.7, 1.4 and 14) were mixed in water at room temperature at 7.2 pH. The white precipitates that formed were collected by centrifugation, thoroughly washed with water for three times to remove unbounded precursors, and analyzed by Fourier transform infrared (FTIR) spectroscopy (Agilent 670) in reflection mode. The spectra were acquired at a resolution of 0.5 cm^−1^ and average of 32 scans.

### Computational Modeling

In order to gain clearer insights into the specific nature of the binding mode of Ca^2+^ and PAA and to better interpret the FTIR data, *ab initio* molecular orbital calculations were also performed. To model PAA we implemented “oligomer approach” by considering two parameters structure of the coordination of metal ion and the vibrational modes of PAA in the presence of Ca^2+^. These results were evaluated by incrementally increasing the oligomer chain length from *N* = 2 to *N* = 8. Minimum energy structure and computed vibrational frequencies converge for chain lengths of N ≥ 4. Hence, we represented PAA using a tetramer; abbreviated as (AA)_4_.

All calculations were performed with the Gaussian 09 suit of programs^40^ with full optimization of the geometries without imposing any constraints. The geometries were optimized until the individual gradients fall below 10^−4^ hartree bohr^−1^ with root mean square force being less than 10^−5^ hartree bohr^−1^. Throughout the study, we used Hartree Fock level theory with 6–31 g(d, p) basis set which allowed rapid evaluations of minimizations. Moreover, this model showed good agreement with the experimental IR spectra of PAA.

To study the coordination of Ca^2+^ with PAA, we used the optimized geometry of the AA_4_ and removed the hydrogen of one of the non-terminal –COOH groups. In order to investigate all of the possible binding modes exhaustively, we positioned the calcium ion along the long axis of the oligomer backbone in eight different positions with identical spacing. We minimized the energy for all of the structures and the lowest energy state is reported. This procedure allowed sampling configurational space effectively and removed the possible bias due to insufficient sampling during minimization. Using the lowest energy state, we positioned the second ion and repeated our procedure to find the minimum energy configuration of oligomer with two metal ions, (AA)_4_Ca_2_.

The same procedure was applied to study the interactions of multiple chains. Initially, we positioned two chains of (AA)_4_ in parallel, with an interchain distance of 6 angstroms and rotate one of them around the other in cylindrical coordinates. We created 10 initial structures equally spaced in angles and minimized each model. The lowest energy structure is reported in this study.

Following the geometry optimization of the two chain complexes, a force constant calculation was also performed to calculate the vibrational frequencies and infrared spectrum. We used HF/6–31 g** and HF/3–21 g* basis sets as they were found to be promising in reproducing experimental vibrational modes and frequencies of PAA coordination with different cations^21^. We scaled the computed frequencies by an empirical correction factor of 0.895 and mimicked the experimental FTIR spectra by fitting intensities with a Gaussian function with a half-band width of 15 cm^−1^.

## Additional Information

**How to cite this article**: Rianasari, I. *et al.* A Chemical Template for Synthesis of Molecular Sheets of Calcium Carbonate. *Sci. Rep.* **6**, 25393; doi: 10.1038/srep25393 (2016).

## Supplementary Material

Supporting Information

## Figures and Tables

**Figure 1:**
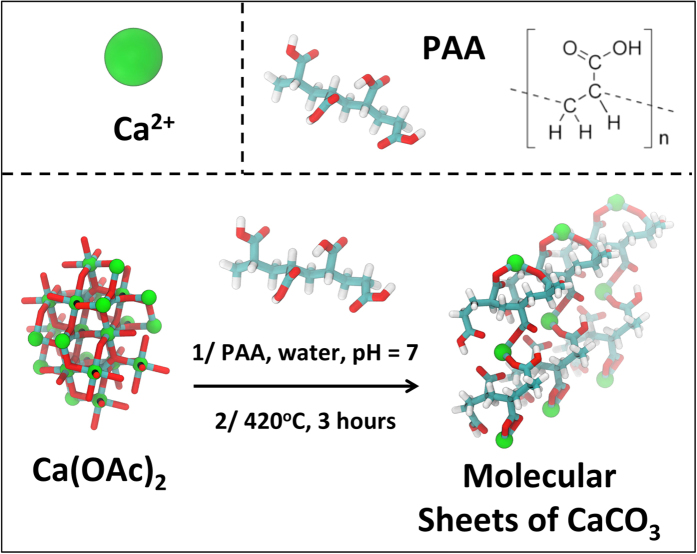
Schematic representation of the molecular sheets of CaCO_3_ formation using a two-step process. A nacre like lamellar structures of molecular sheets of CaCO_3_ interleaved with an organic material, (amorphous carbon) are produced using a formulation of poly(acrylic) acid (PAA) and calcium acetate (Ca-Ac) using a chemical template technique and followed by calcination.

**Table 1:** Δν and Δν_PAA_-Δν obtained for pristine PAA and three different ratios of Ca^2+^/COO^−^ (0.7, 1.4 and 14).

Sample details	Δν(cm^−1^)	Δν_PAA_-Δν
Pristine PAA	224	–
0.7 Molar ratio of Ca^2+^/COO^−^	216	8
1.4 Molar ratio of Ca^2+^/COO^−^	213	11
14 Molar ratio of Ca^2+^/COO^−^	208	16
