# Supplementary material for: A Chemical Template for Synthesis of Molecular Sheets of Calcium Carbonate
Source: Sci Rep. 2016 May 5;6:25393. doi: 10.1038/srep25393 (PMC4857178; doi:10.1038/srep25393)
Supplement: Supporting Information [file srep25393-s1.pdf]

A Chemical Template for Synthesis of Molecular Sheets of Calcium Carbonate

Ina Rianasari[†#] Farah Benyettou,[‡#] Sudhir Kumar Sharma,[†#] Thomas Blanton,[§] Serdal Kirmizialtin‡* and Ramesh Jagannathan†*

SUPPORTING INFORMATION

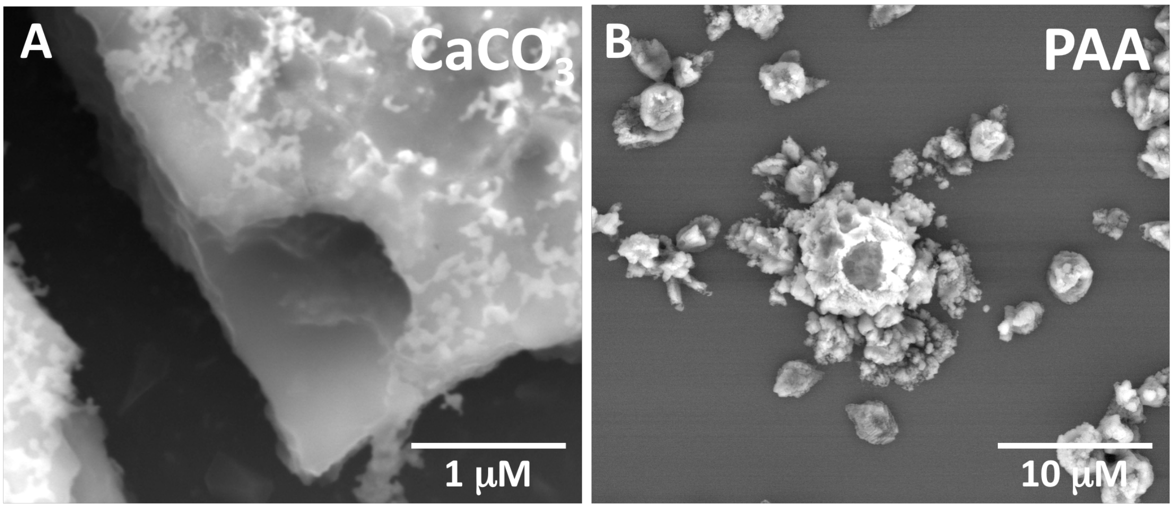

Figure S1. SEM micrographs for A) calcium acetate solution and B) pristine PAA solution.

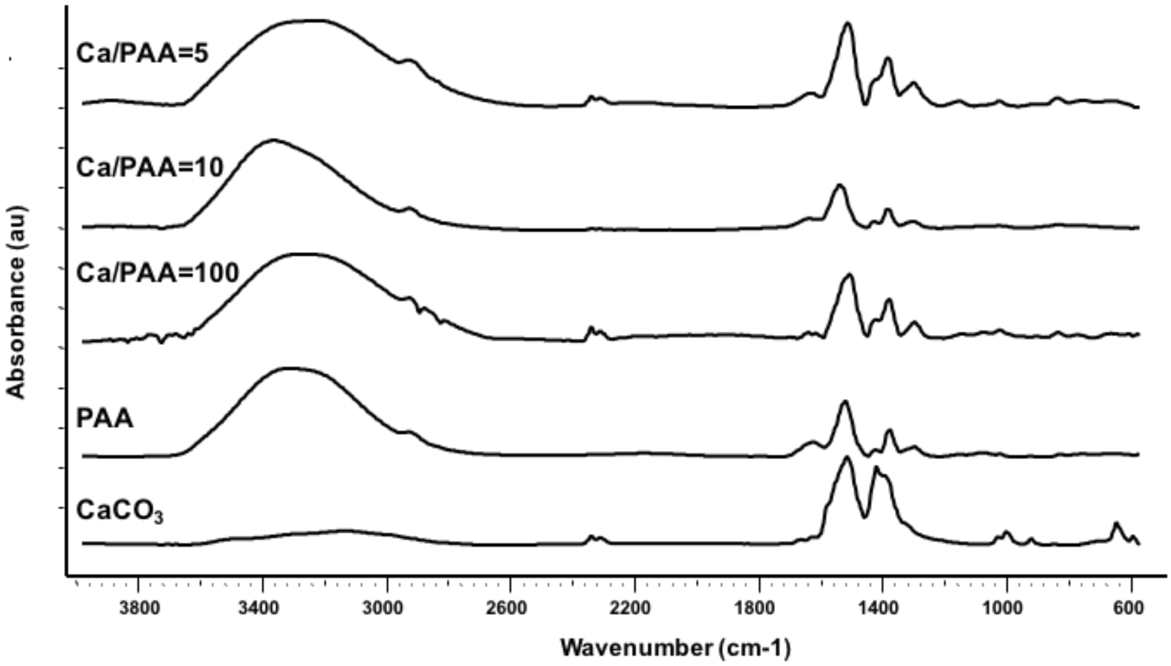

Figure S2. FTIR spectra of dried thin film prepared from the centrifuged and washed complex precipitates on a silicon substrate as a function of the molar ratios of Ca^{2+}/PAA. A) PAA in the absence of Ca^{2+} and in the presence of Ca^{2+} in molar ratios of Ca^{2+}/PAA B) 0.7, C) 1.4, D) 14. No 1035 and 671 cm^{-1} bands were observed in plots B, C and D indicating the absence of free $CaCO_3$ in washed formulations, confirming that the precipitate formation is due to the complexation of the Ca^{2+} and PAA.

Table S1. Standard Enthalpy and Gibbs free energy of each reactant and products forming PAA/Ca^{2+} complexes.

	Gibbs Free Energy (Hartree/Particle)	Enthalpy (Hartree/Particle)
H^+	-0.01	0.00236
$(AA)_4$	-1063.643086	-1063.569753
Ca^{+2}	-676.119268	-676.101696
$(AA)_4^-$	-1062.485464	-1062.41654
$(AA)_4^{-2}Ca^{+2}$	-1739.734987	-1739.663429
$(AA)_4^{-4}(Ca)_2^{+2}$	-2416.049543	-2415.971176

Table S2. Standard Enthalpy of PAA/Ca^{2+} complexes shown in Figure 4

State	Enthalpy (Hartree/Particle)
A-	-1739.663429
B-	-2415.971176
C-	-2803.003159
D-	-4155.176436
E-	-4155.776351
F-	-4155.841636
